# Plectasin, First Animal Toxin-Like Fungal Defensin Blocking Potassium Channels through Recognizing Channel Pore Region

**DOI:** 10.3390/toxins7010034

**Published:** 2015-01-05

**Authors:** Fang Xiang, Zili Xie, Jing Feng, Weishan Yang, Zhijian Cao, Wenxin Li, Zongyun Chen, Yingliang Wu

**Affiliations:** 1 State Key Laboratory of Virology, College of Life Sciences, Wuhan University, Wuhan 430072, China; E-Mails: xiangfang2012@126.com (F.X.); zlxie@whu.edu.cn (Z.X.); jfeng@whu.edu.cn (J.F.); weishanyang@whu.edu.cn (W.Y.); zjcao@whu.edu.cn (Z.C.); wxli@whu.edu.cn (W.L.); 2 Center for BioDrug Research, Wuhan University, Wuhan 430072, China

**Keywords:** plectasin, defensin, potassium channels, Kv1.3 channel, molecular mechanism, functional evolution

## Abstract

The potassium channels were recently found to be inhibited by animal toxin-like human β-defensin 2 (hBD2), the first defensin blocker of potassium channels. Whether there are other defensin blockers from different organisms remains an open question. Here, we reported the potassium channel-blocking plectasin, the first defensin blocker from a fungus. Based on the similar cysteine-stabilized alpha-beta (CSαβ) structure between plectasin and scorpion toxins acting on potassium channels, we found that plectasin could dose-dependently block Kv1.3 channel currents through electrophysiological experiments. Besides Kv1.3 channel, plectasin could less inhibit Kv1.1, Kv1.2, IKCa, SKCa3, hERG and KCNQ channels at the concentration of 1 μΜ. Using mutagenesis and channel activation experiments, we found that outer pore region of Kv1.3 channel was the binding site of plectasin, which is similar to the interacting site of Kv1.3 channel recognized by animal toxin blockers. Together, these findings not only highlight the novel function of plectasin as a potassium channel inhibitor, but also imply that defensins from different organisms functionally evolve to be a novel kind of potassium channel inhibitors.

## 1. Introduction

Mammalian potassium channels are a diverse and ubiquitous family of membrane proteins responsible for the physiological and pathological activities in both excitable and nonexcitable cells [1]. These functional roles have been well elucidated by numerous toxin peptides from venomous animals, such as scorpion, snake, sea anemone, cone snail and bee [2,3,4,5]. These toxins showed high potency and selectivity towards potassium channels with physically blocking the channel pore region explored by docking and molecular dynamic simulations, such as Kv1.3 channel-blocking sea anemone toxin ShK analogues and scorpion toxin HsTx1 analogues [6,7,8,9]. These structurally diverse toxins contain 20–60 amino acid residues, and are cross-linked by two to four disulfide bonds. Similar to animal toxins, defensins, produced by fungus, plants, insects, invertebrate and vertebrate animals, are a kind of antimicrobial peptides stabilized by several disulfide bonds [10,11,12,13]. Despite the similar structures between animal toxins and defensins, whether defensins inhibit potassium channels or not is an open question. Very recently, the answer is given by the first potassium channel-blocking defensin, human β-defensin 2 (hBD2), which was reported to potently and selectively inhibit Kv1.3 channel [14]. Such progress is initiating the discovery process of various defensins as potassium channel blockers from different organisms.

Plectasin, found in *Pseudoplectania nigrella*, is a fungal defensin, which has showed potent antimicrobial activities against various bacterial strains [15,16]. Plectasin has strong affinity to Gram-positive bacterium cell wall subunit Lipid II, inhibits the formation of bacterium cell wall, and present bacterial breeding [17]. Structurally, this 40-amino acid residue peptide can fold into a cysteine-stabilized alpha-beta (CSαβ) structure, resembling potassium channel-blocking scorpion toxins together with same disulfide bridge patterns. These structural commonalities strongly suggest that plectasin might be a toxin-like blocker of potassium channels. Through electrophysiological experiments, plectasin was found to dose-dependently block Kv1.3 channel currents while showed less activity on Kv1.1, Kv1.2, IKCa, SKCa3, hERG and KCNQ channels at the concentration of 1 μΜ. Furthermore, mutagenesis and channel activation experiments indicated that plectasin inhibited Kv1.3 channel currents through interaction with the channel extracellular pore region. Together, these findings first proved plectasin as a new potassium channel blocker, and accelerated the discovery of more potassium channel-blocking defensins from different organisms in the future.

## 2. Results and Discussion

### 2.1. Structural Commonalities between Plectasin and Scorpion Toxins

**Figure 1:**
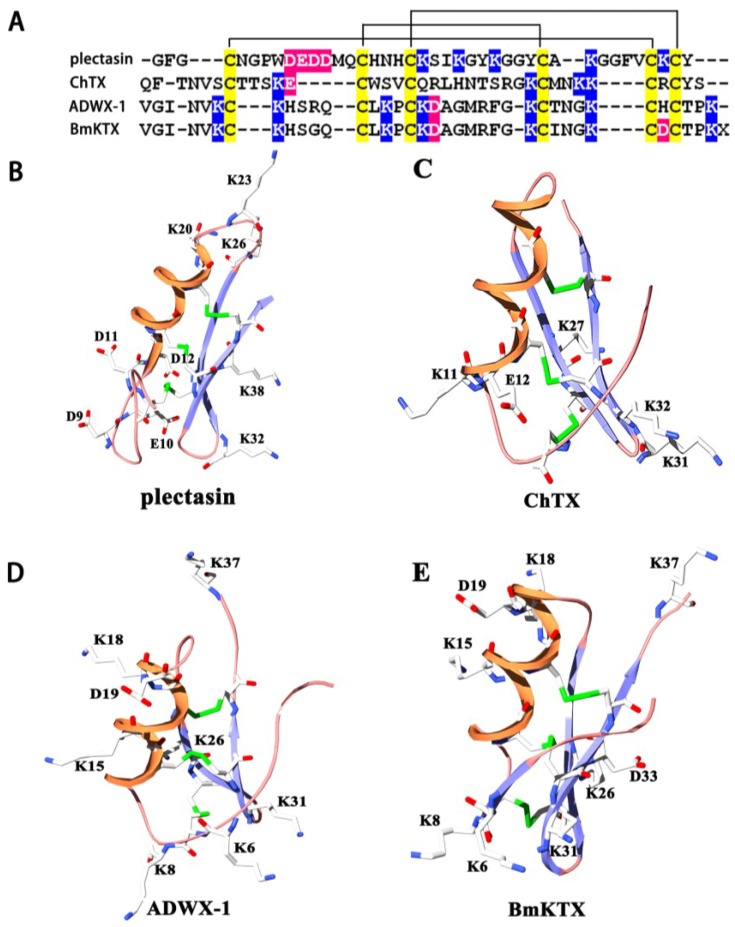
Structural comparison of plectasin and potassium channel-blocking scorpion toxins (**A**) Amino acid sequence alignment analysis of plectasin and scorpion toxins Charybdotoxins (ChTX), Autoimmune Drug from WenXin group (ADWX-1) and BmKTX; (**B**) Acidic and basic residue distribution of plectasin (PDB code: 3E7U); (**C**) Acidic and basic residue distribution of scorpion toxin ChTX (PDB code: 2CRD); (**D**) Acidic and basic residue distribution of scorpion toxin ADWX-1 (PDB code: 2K4U); (**E**) Acidic and basic residue distribution of scorpion toxin BmKTX (PDB code: 1BKT).

Animal toxins blocking the classical potassium channels are well-known basic peptides [2], and plectasin is also a basic peptide (Figure 1A). Although plectasin shows less similar primary structure to those of scorpion toxins acting on potassium channels (Figure 1A), it forms scorpion toxin-like CSαβ structure stabilized by three disulfide bonds (Figure 1B–E). Scorpion toxins are well-known potassium channel blockers with different binding interfaces. For example, scorpion toxin charybdotoxin (ChTX) mainly uses its anti-parallel β-sheet domain to potently inhibit Kv1.x, BKCa and IKCa potassium channels at the nanomolar concentration (Figure 1C) [18,19,20]. Scorpion toxin ADWX-1 also adopts its anti-parallel β-sheet domain to potently inhibit Kv1.x channels (Figure 1D) [21,22]. Different from ChTX and ADWX-1, scorpion toxin BmKTX can use its turn motif between the α-helix and anti-parallel β-sheet domains to recognize Kv1.3 channel (Figure 1E) [23]. Combined other structural types of potassium channel-sensitive hBD2 and animal toxins from snake, bee and sea anemone [2,14], these differential binding interfaces suggest that plectasin might be a toxin-like blocker of potassium channels (Figure 1B–E).

### 2.2. Plectasin Dose-Dependently Blocks Kv1.3 Channel Currents

**Figure 2:**
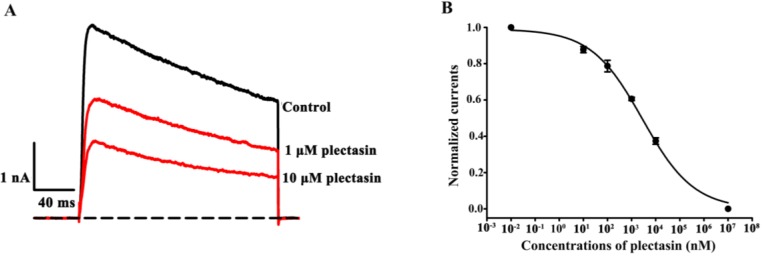
Inhibition of human Kv1.3 channel currents by plectasin (**A**) 39.4% ± 0.9% and 62.6% ± 1.8% of Kv1.3 channel currents by blocked 1 μM and 10 μM plectasin, respectively; (**B**) Average normalized current inhibition by various concentrations of plectasin for Kv1.3 channel. Hill equation fitting gives an IC_50_ value of 2.8 ± 0.6 μM.

Among the potassium channels, Kv1.3 channel shows an unusually broad sensitivity to peptide toxins from the venomous animals [2,24], and such sensitivity was also recently revealed by hBD2 [14]. Therefore, we first investigated the effect of plectasin on Kv1.3 channels, which were transfected and expressed in HEK293 cells. As shown in Figure 2A, 1 μM and 10 μM plectasin were able to inhibit 39.4% ± 0.9% and 62.6% ± 1.8% of Kv1.3 channel currents, respectively (Figure 1A). Furthermore, the concentration-dependent experiments showed that plectasin blocked Kv1.3 channel currents with an IC_50_ value of 2.8 ± 0.6 μM (Figure 2B). These data indicated that plectasin was a novel blocker of Kv1.3 channel. Although plectasin shows weaker affinity towards Kv1.3 channel than some animal toxins, including toxin analogs in the preclinical and clinical trials [4,25,26], it would simultaneously exert both Kv1.3 channel-blocking and antimicrobial effects.

Presently, there is less information on the survival and evolution relationships between plectasin and animal toxins according to the best of our knowledge. Therefore, the common potassium channel-blocking activities between plectasin and animal toxins would make following open questions: (1) Why does plectasin evolve to be a potassium channel blocker? (2) Is there potential evolutionary relationship between plectasin and animal toxins?

### 2.3. Plectasin Shows Much Weaker Activities on Other Potassium Channel Subtypes

**Figure 3:**
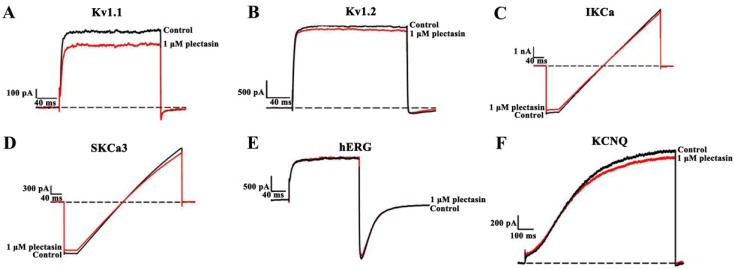
The plectasin interaction with other potassium channels (**A**) 1 μM plectasin blocked 16.4% ± 3.1% of Kv1.1 channel currents; (**B**) 1 μM plectasin blocked 4.0% ± 1.3% of Kv1.2 channel currents; (**C**) 1 μM plectasin blocked 7.0% ± 2.1% of IKCa channel currents; (**D**) 1 μM plectasin blocked 5.8% ± 1.8% of SKCa3 channel currents; (**E**) 1 μM plectasin blocked 4.1% ± 0.9% of hERG channel currents; (**F**) 1 μM plectasin blocked 3.4% ± 1.2% KCNQ channel currents.

Based on the interaction between plectasin and Kv1.3 channel, we further investigated the possible binding of plectasin to additional potassium channels, which were also transfected and expressed in HEK293 cells. Through the electrophysiological experiments, it was found that 1 μM plectasin could block 16.4% ± 3.1%, 4.0% ± 1.3%, 7.0% ± 2.1%, 5.8% ± 1.8%, 4.1% ± 0.9% and 3.4% ± 1.2% of potassium currents mediated by Kv1.1, Kv1.2, IKCa, SKCa3, hERG and KCNQ channels, respectively. These data indicated plectasin was a much weaker blocker for these tested potassium channel subtypes than Kv1.3 channel (Figure 3), which again demonstrated the unusual sensitivity of Kv1.3 channel towards defensins and various toxins [2,14,21,23,24]. In view of physiological and pathological importance of Kv1.3 channels in human lymphocyte [4,5], the selective binding of plectasin towards Kv1.3 channel would play a critical role in the immunomodulatory functions when using plectasin against microbes in the infected skin or other tissues.

### 2.4. Kv1.3 Channel Pore Region Is Plectasin-Interacting Site

**Figure 4:**
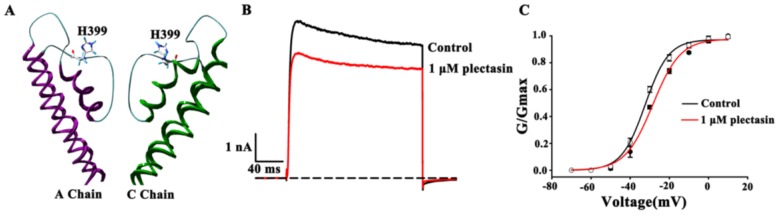
The molecular mechanism of plectasin binding to Kv1.3 channel (**A**) Distribution of His399 residues in the pore region of the modeled Kv1.3 channel; (**B**) 1 μM plectasin blocked 21.2% ± 3.2% of Kv1.3-H399A channel currents; (**C**) The effect of plectasin on activation G-V curves of Kv1.3 channel. The V_50_ values are −32.4 ± 0.5 mV and −28.4 ± 0.8 mV before and after applying 1 μM plectasin.

The extracellular pore region of Kv1.3 channel is the binding site of hBD2 and various toxins [14,22]. Plectasin, as a toxin-like blocker would also likely recognize channel vestibule for inhibiting Kv1.3 channel currents. In order to test this hypothesis, His399 residues, near the channel selectivity filter, is selected (Figure 4A) since they play essential roles in the binding of hBD2 and different toxins [14,22]. The mutant Kv1.3-H399A channels were then constructed and tested by plectasin binding. In comparison with 39.4% ± 0.9% inhibition of wild-type Kv1.3 channel currents by 1 μM plectasin, there was 21.2% ± 3.2% inhibition of mutant Kv1.3-H399A channels at the identical concentrations (Figure 4B). These differential activities of plectasin acing on wild-type and mutant Kv1.3 channels indicate that plectasin is able to inhibit Kv1.3 channel through interacting with channel extracellular pore region.

In order to further verify the mechanism of plectasin blocking Kv1.3 channel, the channel conductance-voltage relationship (G-V) curves were measured in the absence and presence of plectasin. As show in Figure 4C, 1 μM plectasin did not significantly shift the G-V curve of Kv1.3 channel, and V_50_ values were −32.4 ± 0.5 mV and −28.4 ± 0.8 mV in the absence and presence of plectasin, respectively (Figure 4C). Such effect was similar to that of Kv1.3 channel-blocking ADWX-1 toxin without obviously changing the channel G-V curve [27]. Therefore, plectasin is an animal toxin-like blocker interacting with the extracellular pore region of potassium channels.

## 3. Materials and Methods

### 3.1. Peptides and Potassium Channels

Plectasin was purchased from Peptide Institute (Osaka, Japan). The pRc/CMV-hKv1.3 vector was kindly provided by Prof. Stephan Grissmer (University of Ulm, Ulm, Germany) and Prof. Olaf Pongs (Zentrum für Molekulare Neurobiologie der University Hamburg, Hamburg, Germany). The cDNAs encoding mouse Kv1.1, human Kv1.2, human IKCa, human SKCa3, hERG and human KCNQ channels (from Prof. Stephan Grissmer, University of Ulm, Ulm, Germany and Prof. George Chandy, University of California, Irvine, CA, USA) were subcloned into the vector of pIRES2-EGFP (TaKaRa Clontech, Mountain View, CA, USA) for coexpression with GFP. His^399^ on the pore of wild type Kv1.3 plasmid were mutanted into Ala with QuikChange Site-Directed Mutagenesis Kit (Agilent Stratagene, Santa Clara, CA, USA). The mutant was verified by DNA sequencing before being used.

### 3.2. Cell Culture

HEK293 cells were cultured Dulbecco’s modified Eagle’s medium (Invitrogen) with 10% heat-inactivated fetal calf serum supplemented with penicillin (100 units/mL) and streptomycin (100 μg/mL) in a humidified 5% CO_2_ incubator at 37 °C. Plasmids transfected into HEK293 cells using FuGENE transfection reagent (Shanghai Roche Pharmaceuticals Ltd., Shanghai, China) following the manufacturer’s instructions. Potassium currents were recorded 1 to 3 days after transfection and cell culture and positive cells were selected based on the presence of GFP fluorescence.

### 3.3. Electrophysiological Recordings

Electrophysiological experiments were carried out at 22–25 °C using the patch-clamp whole-cell recording mode according to our previously protocols [3,14,18,21,22,23,24,27]. For the Kv1.3 channel, HEK293 cells were bathed with mammalian Ringer’s solution: 5 mM KCl, 140 mM NaCl, 10 mM HEPES, 2 mM CaCl_2_, 1 mM MgCl_2_, 10 mM D-glucose, pH 7.4 with NaOH. When Plectasin was applied, 0.01% BSA was added to the Ringer’s solution. A multichannel microperfusion system MPS-2 (INBIO Inc., Wuhan, China) was used to exchange the external recording bath solution. The internal pipette solution contain 140 mM KCl, 1 mM MgCl_2_, 1 mM EGTA, 1 mM Na_2_ATP, 5 mM HEPES (pH 7.4 with KOH). For the recording of calcium-activated potassium channels, the bath contained 130 mM sodium aspartate, 30 mM potassium aspartate, 2 mM CaCl_2_, 1 mM MgCl_2_, and 10 mM HEPES (pH adjusted to 7.2 with NaOH). The pipette solution contained 145 mM potassium aspartate, 8.7 mM CaCl_2_, 2 mM MgCl_2_, 10 mM EGTA, and 10 mM HEPES (pH adjusted to 7.2 with KOH) to achieve an intracellular free Ca^2+^ concentration of 1 μM. All channel currents were elicited by depolarizing voltage steps of 200 ms from the holding potential −80 mV to +50 mV. Membrane currents were measured with an EPC 10 patch clamp amplifier (HEKA Elekt-ronik, Lambrecht, Germany) interfaced to a computer running acquisition and analysis software.

## 4. Conclusions

Based on the structural commonalties between plectasin and potassium channel-blocking animal toxins, we found that plectasin was the first potassium channel blocker from a fungus. More interestingly, plectasin was a selective blocker of Kv1.3 channel with IC_50_ value of 2.8 ± 0.6 μM. Similar to the classical potassium channel-blocking animal toxins, plectasin was able to inhibit potassium channel currents through interaction with channel extracellular pore region. Such findings would prompt the in-depth investigation of defensins as potential potassium channel inhibitors from different organisms in the future. Furthermore, the evolutionary role of plectasin as a novel potassium channel blocker will become an interesting question.
